# Assessment of the genomic variation in a cattle population by re-sequencing of key animals at low to medium coverage

**DOI:** 10.1186/1471-2164-14-446

**Published:** 2013-07-04

**Authors:** Sandra Jansen, Bernhard Aigner, Hubert Pausch, Michal Wysocki, Sebastian Eck, Anna Benet-Pagès, Elisabeth Graf, Thomas Wieland, Tim M Strom, Thomas Meitinger, Ruedi Fries

**Affiliations:** 1 Chair of Animal Breeding, Technische Universität München, Liesel-Beckmann-Strasse 1, Freising, 85354, Germany; 2 Institute of Human Genetics, Helmholtz Zentrum München, German Research Center for Environmental Health, Neuherberg, 85764, Germany; 3 Institute of Human Genetics, Technische Universität München, Munich, 81675, Germany

**Keywords:** Next-generation sequencing, Low-coverage, Genotyping by sequencing, Variant annotation

## Abstract

**Background:**

Genome- and population-wide re-sequencing would allow for most efficient detection of causal trait variants. However, despite a strong decrease of costs for next-generation sequencing in the last few years, re-sequencing of large numbers of individuals is not yet affordable. We therefore resorted to re-sequencing of a limited number of bovine animals selected to explain a major proportion of the population's genomic variation, so called key animals, in order to provide a catalogue of functional variants and a substrate for population- and genome-wide imputation of variable sites.

**Results:**

Forty-three animals accounting for about 69 percent of the genetic diversity of the Fleckvieh population, a cattle breed of Southern Germany and Austria, were sequenced with coverages ranging from 4.17 to 24.98 and averaging 7.46. After alignment to the reference genome (UMD3.1) and multi-sample variant calling, more than 17 million variant positions were identified, about 90 percent biallelic single nucleotide variants (SNVs) and 10 percent short insertions and deletions (InDels). The comparison with high-density chip data revealed a sensitivity of at least 92 percent and a specificity of 81 percent for sequencing based genotyping, and 97 percent and 93 percent when a imputation step was included. There are 91,733 variants in coding regions of 18,444 genes, 46 percent being non-synonymous exchanges, of which 575 variants are predicted to cause premature stop codons. Three variants are listed in the OMIA database as causal for specific phenotypes.

**Conclusions:**

Low- to medium-coverage re-sequencing of individuals explaining a major fraction of a population's genomic variation allows for the efficient and reliable detection of most variants. Imputation strongly improves genotype quality of lowly covered samples and thus enables maximum density genotyping by sequencing. The functional annotation of variants provides the basis for exhaustive genotype imputation in the population, *e.g.*, for highest-resolution genome-wide association studies.

## Background

Association studies for the mapping of quantitative trait loci (QTL) and genetic evaluation in cattle is presently based on high-density SNP marker sets. The DNA variants that are causally involved in QTL variation are not necessarily among these markers. Since linkage disequilibrium of markers and causal variants is not complete, marker-based QTL mapping and evaluation are consequently not maximally efficient [1,2]. Maximal efficiency can only be achieved if all variable positions in the genome are assessed by whole-genome re-sequencing [3].

Although the advent of the next generation sequencing technology led to a dramatic reduction of sequencing costs, whole-genome sequencing of thousands of animals per population is still not affordable. Scheet and Stephens [4] proposed to sequence a subset of individuals and to impute the missing genotypes in the general population *via* genotypes obtained by a SNP chip. The sequenced animals are ideally key ancestors of the population, *i.e.* unrelated individuals with a maximal contribution to the genomic variation of the modern population [5,6].

The feasibility and affordability of a whole-genome sequencing project also depends on the coverage required for efficient variant detection. A pilot of the Human 1000 Genomes Project, consisting of sequencing 179 individuals with a coverage of 3.6x per individual only, allowed to catalogue the vast majority of variation in this sample [7]. The low coverage prevented immediate and reliable variant calling at many sites. However, multi-sample analysis and imputation provided a means of highly accurate and complete genotyping, compensating for the low coverage [8,9].

The promising results of the first phase Human 1000 Genomes Project prompted us to apply low- to medium-coverage sequencing to key ancestors and some contemporary animals of a cattle population. Imputation within the sample of 43 sequenced animals and subsequent genotype imputation allowed the identification of more than 17 million variable sites, most of them novel, with high confidence.

## Results and discussion

### Selection of key animals for re-sequencing

**Figure 1:**
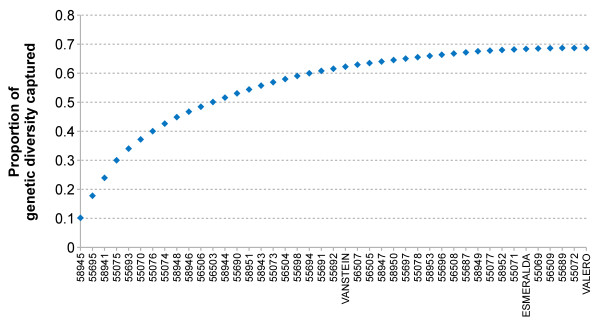
**Proportion of genetic diversity captured by re-sequencing of 43 key animals of the Fleckvieh population.** Forty-three animals chosen for re-sequencing are ordered based on their genetic contribution to the current Fleckvieh population. Among the re-sequenced animals is a trio including the previously re-sequenced *VANSTEIN*[36], his mating partner *ESMERALDA* and their common progeny *VALERO*.

Re-sequencing of a panel of optimally selected 43 key ancestors of the Fleckvieh population would enable to capture ~76 percent of the genetic diversity of the current population. However, obtaining DNA samples of key ancestors is often difficult. The animals died decades ago and semen samples are not always available. Thus, the actual 43 animals chosen for re-sequencing account for about 69 percent of the genetic diversity only. However, the twelve most influential animals account for ~50 percent of the genetic diversity of the entire population (Figure 1). The set of selected animals comprises one sire-dam-offspring trio and totally eight parent-offspring duos (Additional files 1 and 2).

### Re-sequencing data

Close to ten billion paired-end reads of 36, 76, 96, 100 and 101 bases were obtained for the 43 animals on Illumina GA IIx and HiSeq2000 instruments. From 90.06 percent to 98.35 percent of the reads were mapped to the 30 chromosome scaffolds (autosomes 1 – 29, X-chromosome) of the bovine reference genome assembly UMD3.1 [10] encompassing 2.66 billion bases. Not considering PCR duplicates, that amounted from 3.59 to 15.55 percent (average 7.42 percent) of the reads, the coverage ranged from 4.19 to 24.98 reads per position, with an average of 7.46 reads for the 43 animals (Additional file 3). The re-sequencing data have been contributed to the 1000 bull genomes project [11].

### Detection of single nucleotide variants and short insertions/deletions

**Figure 2:**
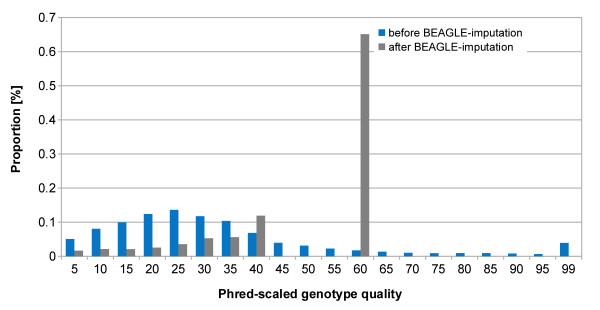
**Distribution of genotype-qualities before and after *Beagle *imputation.** The quality of each genotype was specified by its phred-scaled likelihood. The maximun score was restricted to 60 after *Beagle* imputation at 15,438,621 biallelic sites.

Multi-sample variant detection [12] allowed the calling of genotypes at 17,170,787 positions in each animal, meaning that at least one individual showed a deviation from the UMD3.1 reference sequence at each of these positions. In the case of missing or insufficient coverage, the most likely genotype was called based on the allele frequency at this site. *Beagle*[13] haplotype phasing and imputation raised the percentage of genotypes with phred-scaled quality scores greater than 10 from 94.92 (before imputation) to 98.34 (Figure 2). Most of the variants (89.91 percent) were biallelic single nucleotide polymorphisms (SNPs) (Table 1). Insertion / deletion variants (InDels) amounted to 9.94 percent of the variants (54.45 percent deletions, 45.55 percent insertions). The remaining 0.15 percent were triallelic variants. Of the SNPs detected in this study, 67.95 percent represent newly identified variants; the remaining 32.05 percent are listed in build 133 of dbSNP. The rate of transitions to transversions (Ti/Tv) amounts to 2.21, which is within the range observed in previous whole-genome sequence studies [8]. The number of biallelic SNPs per animal ranged from 5,885,050 to 6,366,501 and did not depend on the coverage (Additional file 4). The proportion of biallelic SNP calls at sites without coverage ranged from 0.54 percent to 9.6 percent in the 43 animals (average: 3.47 percent).

**Table 1:** Counts of variants per chromosome

**Chromosome**	**Biallelic**	**Triallelic**	**InDels**
1	953,116	1,594	106,808
2	765,523	1,540	86,271
3	689,653	1,061	74,819
4	727,730	1,059	81,188
5	711,291	1,236	78,518
6	715,956	1,306	81,170
7	647,618	944	72,861
8	662,730	929	73,740
9	605,302	884	69,531
10	628,109	968	68,543
11	594,105	1,102	64,693
12	658,305	1,117	70,418
13	467,872	977	50,653
14	496,824	852	54,644
15	563,600	736	60,307
16	479,831	785	52,312
17	454,188	722	51,040
18	378,391	604	40,948
19	370,300	787	39,877
20	449,443	744	49,207
21	410,810	612	44,478
22	335,787	520	36,763
23	420,876	676	42,587
24	371,270	632	40,919
25	269,464	430	27,099
26	316,133	507	34,077
27	287,872	470	32,574
28	290,731	482	31,154
29	350,281	665	36,219
X (non PAR)	285,956	486	44,949
X (PAR)	79,554	119	8,253

Multi-sample variant calling was performed as described in Methods. The X chromosome was divided into a pseudoautosomal region (PAR) and a non-pseudoautsomal region (PAR) (see Additional file 9).

Variants were submitted to dbSNP (National Center for Biotechnology Information, National Library of Medicine, Bethesda, MD) under handle ID TZ_TUM (dbSNP Build ID: B138) and will be available from the NCBI SNP database website upon the next build in March 2013.

### Evaluation of variant-calling

**Figure 3:**
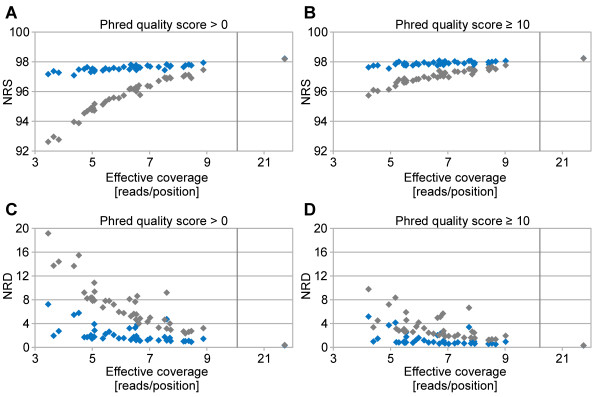
**Comparison of re-sequencing-derived with genotypes resulting from array-based genotyping.** Non-reference sensitivity (NRS), considering all genotypes (phred-scaled quality score ≥ 0) **(A)**. NRS, phred-scaled quality score ≥ 10 **(B)**. Non-reference discrepancy (NRD), phred-scaled quality score ≥ 0 **(C)**. NRD, phred-scaled quality score ≥ 10 **(D)**. The grey and the blue symbols represent the NRS and NRD before and after *Beagle* imputation, respectively.

We compared array-called genotypes with sequence-derived genotypes at corresponding positions and calculated the non-reference sensitivity (NRS) and the non-reference discrepancy (NRD) rates [8] to assess the sensitivity and specificity, respectively, of the variant calling. Both measures depend on the coverage, on whether or not *Beagle* imputation is applied and on the minimal phred-scaled likelihood requirement (Figure 3). The effects of *Beagle* imputation and minimal phred score requirement are most prominent if the coverage is below 7x. The NRD rate for the animal with the lowest coverage (4.17x) was 9.78 percent before and 5.17 percent after *Beagle* imputation. Highest NRS values at comparably low NRD values are achieved after *Beagle* imputation and after removal of genotypes with phred scores below 10.

### Functional annotation of variants

**Figure 4:**
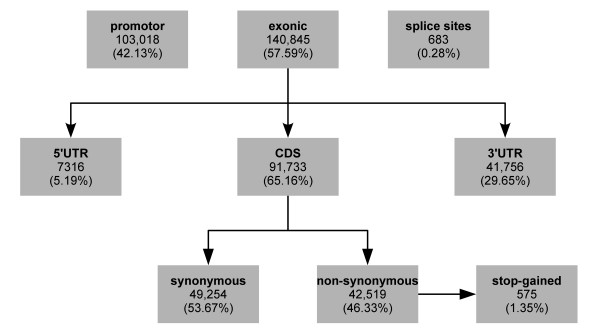
**Functional annotation of variants.** Functionally annotated biallelic SNPs and InDels in 18,444 genes (see Methods) including 1,000 bases of promotor sequence.

The annotated sequence, including 1,000 basepairs of putative promotor sequence, encompasses 18,444 genes and 55,753,651 basepairs of sequence and exhibits a total of 244,546 variants (Figure 4). The coding sequence is affected by 91,733 exchanges, 42,519 (46.33 percent) of which are non-synonymous. Kawahara-Miki et al. [14] reported a considerably higher rate (57 percent) for a Japanese animal. Our result is more in line with the findings by Stothard et al. [15] and Zhan et al. [16] of 42 percent of non-synonymous exchanges in an Angus and two Holstein animals. Fifty-eight percent of the non-synonymous substitutions are predicted to be “probably damaging” or “possibly damaging” (Additional file 5) by PolyPhen-2 and 575 (1.35 percent) are predicted to cause a premature stop codon, similar to reported findings [15,16]. Each of the putative stop codon introducing variants needs to be scrutinized by manual examination of the reads, the re-annotation of the affected genes and Sanger sequencing. *Bona fide* stop codon causing variants need to be typed in a representative sample. Absence of homozygosity may reveal undetected recessive disorders with nondescript or embryonically / fetally lethal phenotypes, with the latter manifesting themselves as reduced fertility [17,18].

**Figure 5:**
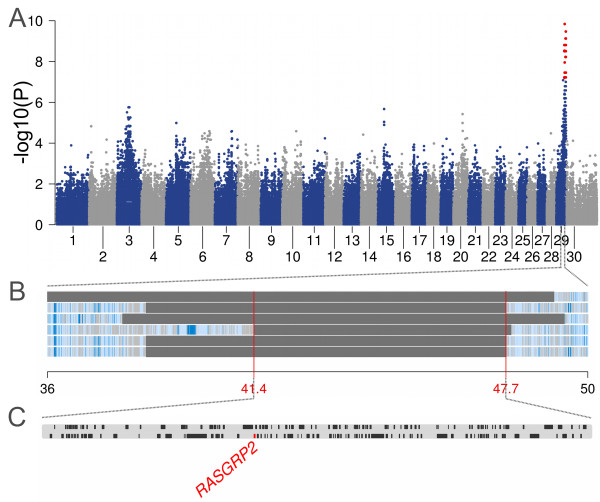
**Mapping of a bleeding disorder in the Fleckvieh population.** Association of 652,856 SNPs with the affection status of 43 re-sequenced and six thrombopathic animals **(A)**. The affected animals were reported to the Clinic for Ruminants, Faculty of Veterinary Science, University of Munich). P values were obtained using Fisher exact tests of allelic association. Autozygosity mapping in six thrombopathic animals revealed a common segment of extended homozygosity (41.3 – 47.7 Mb) **(B)** including the position of *RASGRP2***(C)**.

The catalogue of annotated variants allows to examine whether any of the causal variants for bovine Mendelian traits / disorders listed in OMIA – Online Mendelian Inheritance in Animals [19] is segregating in the re-sequenced sample of Fleckvieh and therefore in the entire Fleckvieh population. The UMD3.1 reference coordinates could be determined for 59 of the OMIA entries (Additional files 6 and 7). The 2bp deletion in *MOCS1* responsible for Arachnomelia (OMIA 001541–9913; [20]) could be detected in a known heterozygous carrier of the defective variant. An amino acid exchange in *RASGRP2* has been proposed to be the causal variant for a bleeding disorder in a Simmental animal (Thrombopathia, OMIA 001003–9913; [21]). Eight of the re-sequenced animals carry the variant. It turns out that there are indeed animals in the Fleckvieh population being affected with the bleeding disorder. A genome-wide association study with six thrombopathic animals as case group and 43 re-sequenced animals as controls yielded a strong association signal on chromosome 29 (Figure 5). Subsequent autozygosity mapping in the affected animals revealed a common 6.3 Mb segment of extended homozygosity encompassing the location of *RASGRP2* and corroborating recessive inheritance. Sanger sequencing confirmed that all thrombopathic animals are homozygous for the pertinent amino acid exchange (c.701T > C, p.L234P, Chr29:43599204). Among the affected animals are descendants of a re-sequenced bull. This bull did not appear to carry the variant. However, the relevant position is covered by two reads only, and after *Beagle* imputation, we could indeed observe heterozygosity that was subsequently confirmed by Sanger sequencing. This exemplifies both the power of re-sequencing key ancestors for the detection of genetic disorders and the importance of the imputing step at lowly covered sites. The Fleckvieh breed exhibits a recessively inherited red and white coat (relative to “dominant black”) and is thus expected to be homozygous for the “red factor” causing deletion in the *MC1R* gene (OMIA 001199–9913; [22]). All but one animal are homozygous. The only heterozygous animal, carrying both the deletion and the wild type allele is red and white. The reference sequence, derived from red and white Hereford animals [23], also contains the wild type allele of the *MC1R* gene. A deletion variant of *PMEL* (OMIA 001545–9913) causing coat color dilution segregates in the re-sequenced animals at a frequency of 0.22 without apparent phenotypic effect. However, carriers of the variant allele are reported to produce progeny exhibiting a “diluted black” phenotype when crossed with black and white animals that carry the “dominant black” allele of *MC1R.* Thus, our findings are in line with Schmutz and Dreger's [24] discovery of interacting *MC1R* and *PMEL* alleles.

There are only few reports on the detection of causal variants for quantitative traits in cattle, so called QTNs [25]. A lysine-to-alanine substitution in acylCoA:diacyglycerol acyltransferase (*DGAT1*; c.694_695GC > AA, p.A232K, Chr14:1802265–1802266) has a major effect on the fat content of milk and milk yield [26,27].The frequency of the milk-fat-enhancing and milk-yield-lowering lysine-allele is 0.02 in the re-sequenced animals. A variant in the growth hormone receptor gene (*GHR),* causing a phenylalanine-to-tyrosine substitution (c.836T > A, p.F279Y, Chr20:31909478) that affects milk yield and composition [28,29] is segregating in the re-sequenced animals, with the milk-yield-enhancing and content-lowering tyrosine-allele amounting to a frequency of 0.05. A missense mutation in the gene encoding ATP-binding cassette, sub-family G (WHITE), member 2 (*ABCG2*; c.1742A > C, p.Y581S, Chr6:38027010) affecting milk yield and composition [30] is not segregating in the re-sequenced Fleckvieh animals, *i.e*., they are fixed for the milk yield-enhancing allele. Allelic variation of the prolactin receptor gene (*PRLR;* c.54_55GT > AC, p.S18N, Chr20:39115344–39115345) affects protein and fat yield [29]. The asparagine-allele is yield-enhancing and has a frequency of 0.20 in the re-sequenced animals. Eberlein *et al.*[31] have shown association of a non-synonymous substitution in the G subunit of the non-SMC condensin complex (*NCAPG*, c.1326T > G, p.I442M, Chr6:38777311) with fetal growth. Our re-sequencing reveals the allele leading to reduced fetal growth at a frequency of 0.29. A recently postulated functional polymorphism close to the gene for pleiomorphic adenoma gene 1 (*PLAG1*) affecting stature [32,33] could not be assessed since it is located in a not-sequenced gap of the UMD3.1 reference sequence.

## Conclusions

Next-generation sequencing of key ancestors of a cattle breed and multi-sample calling as recommended by Nielsen [9] allowed the reliable detection of more than 17 million variable positions in the bovine genome, more than eleven million of them novel. Thus, this study significantly adds to the endeavour of sampling the genomic variation in *Bos taurus*. We show that genotype imputation within the re-sequenced animals enhances genotype quality considerably, particularly in lowly covered samples.

The re-sequenced animals represent 69 percent of the genetic diversity of the Fleckvieh population. We can expect the majority of causal variants, at least the not very rare ones, to be segregating among the sequenced animals. If a causal variant can be pinpointed to a specific haplotype as a result of genome-wide association studies, and if the haplotype is present in the sequenced ancestors, identification will often be straightforward. Furthermore, inspecting already identified causal variants, such as listed in Online Mendelian Inheritance of Animals (OMIA, [19]), allows to readily monitor the population for genetic disorders. Since false positives are of no concern in this process, unfiltered variants should be considered and any match be confirmed by Sanger sequencing. Variant annotation led to the detection of 575 putative premature stop codons. Each of the underlying variants needs to be scrutinized with regard to possible annotation errors and to be confirmed by Sanger sequencing. Premature stop codons often represent loss of function mutations with potentially severe consequences such as embryonic lethality in the homozygous state. Thus, re-sequencing of key ancestors and functional annotation of variants should allow for a proactive management of genetic disorders.

The genotypes at more than 17 million positions can now be imputed in any animal of the Fleckvieh breed with array-derived medium or high density genotypes. Imputation accuracy should be high, at least for loci with minor allele frequencies > 0.05, since the re-sequenced animals, as key ancestors, are highly representative for the population [34]. However, increasing the number of re-sequenced animals will be necessary for maximal imputation accuracy for loci with lower minor allele frequency. One can now envisage to perform genome-wide association studies based on re-sequencing derived and imputed genotypes to achieve maximal resolution of QTL mapping and the perspective of direct identification of causal variants. The imputed genotypes will also be the basis for more accurately predicting genomic values of selection candidates [3].

## Methods

### Animal ethics statement

Semen samples were collected by approved commercial artificial insemination stations as part of their regular breeding and reproduction measures in cattle industry. The collection of blood samples was carried out by trained veterinarians during treatment of affected animals following standard veterinary protocols in Germany. No ethical approval was required for this study.

### Identification of key animals for sequencing

Genotype and pedigree data were available for 3645 animals representing an informative subset of the current German Fleckvieh population [35]. The genotyped animals were born between 1966 and 2009. Individuals accounting for a major fraction of the genetic diversity of the available sub-population were considered as key animals. Key animals were identified iteratively with $p_{m}=A_{m}^{-1}*c_{m}$[5], where *A* is the numerator relationship matrix of *m* selected animals with pedigree information lasting back to 1920, *c* is a vector representing the average relationship of the selected animals to the entire population and *p* is a vector with the proportion of genetic diversity captured by *m* animals.

### Generation of sequence data

Genomic DNA was prepared from semen straws and blood samples following standard protocols using proteinase K digestion and phenol-chloroform extraction. DNA-concentration was set to 250 ng/μl. Paired-end libraries were prepared using the paired-end TruSeq DNA sample prep kit (Illumina inc., San Diego, CA, USA) and sequenced using Illumina GA IIx and HiSeq 2000 instruments (Illumina inc., San Diego, CA, USA). The read length ranged from 76 to 101 bp (Additional file 8). Paired-end reads with a length of 36 bp were available for one bull from a former study [36]. The resulting reads were processed with the Illumina BaseCaller during the sequencing step. The alignment of the reads to the University of Maryland reference sequence (UMD3.1) [10] was performed with BWA (version 0.6.1-r104) [37] using default parameters. The resulting per individual SAM files were converted into BAM files with SAMtools (version 0.1.18) [38]. Duplicate reads were identified and marked with the *MarkDuplicates* command of Picard [39].

### Variant-calling and imputation

Calling of variants including short insertions and deletions (InDels) was performed for the 43 sequenced animals simultaneously using *mpileup* of SAMtools (version 0.1.18) along with BCFtools [38]. The *extended base alignment quality* (BAQ) option was applied to scrutinize variant calling around InDels. The X chromosome was divided into a recombining pseudoautosomal region (PAR), and a non-pseudoautosomal region (non PAR) (Additional file 9). Read duplicates (see above) and positions with a coverage of more than 400 reads were not considered in variant calling. *Beagle* phasing and imputation [13] within the 43 sequenced animals was used to improve the primary genotype calling by SAMtools. The resulting variants were compared with those listed in the latest version of dbSNP (build 133; http://ftp://ftp.ncbi.nlm.nih.gov/snp/organisms/cow_9913/chr_rpts/).

### Evaluation of variant-calling

**Figure 6:**
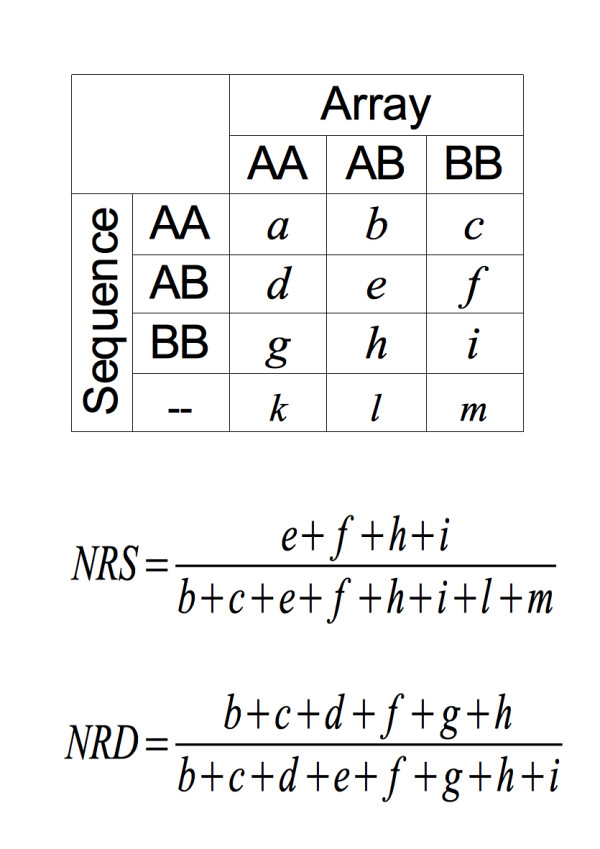
**Non-reference sensitivity (NRS) and non-reference discrepancy rate (NRD). (A)** Sequence-derived genotypes were compared with array-based genotypes in order to measure the accuracy of genotype calls. **(B)** The mathematical formula for calculation of NRS and NRD, based on DePristo *et al. *[8].

All 43 re-sequenced animals were genotyped with the Illumina BovineHD BeadChip®. The average per individual call rate was 99.26 percent. The array-derived genotypes were compared with the sequence-derived genotypes at in average 46,268 positions on chromosome 1 which we considered as representative for the autosomal genome. Non-reference sensitivity (NRS) and non-reference discrepancy (NRD) rates were calculated as proposed by DePristo *et al.* (2011) [8] and as shown in Figure 6.

### Variant annotation

The functional effects of unfiltered variants were analysed based on the annotation of the UMD3.1 assembly of the bovine genome with 21,364 protein coding transcripts [40]. Gene models leading to one or more in-frame stop codon were not considered, restricting the variant annotation to 18,444 protein coding transcripts (one per gene), including 32 manually annotated or re-annotated transcripts (Additional file 10). Positioning and analysis of variants in the promotor (defined to encompass 1,000 bp upstream of the transcription start), in the 5'-UTR, in the amino-acid coding region, in splice sites and the 3'-UTR were performed using a database of unfiltered variants set up in MySQL and by custom scripts written in Python. *PolyPhen2-2 v.2.2.2*[41] was applied for predicting effects of non-synonymous mutations.

### Availability of supporting data

The identified variants were submitted to the Database of Single Nucleotide Polymorphisms (dbSNP). Bethesda (MD): National Center for Biotechnology Information, National Library of Medicine. (dbSNP Build ID: {138}). Available from: http://www.ncbi.nlm.nih.gov/SNP/

## Competing interests

The authors declare that they have no competing interests.

## Authors’ contributions

RF, TMS and TM conceived the study and participated in its design and coordination. MW, ABP and EG performed the sample and library preparation and carried out the sequencing experiments. SJ, BA, HP, SE, TW, TMS and RF analysed the data. SJ and RF drafted the manuscript. All authors read and approved the final manuscript.

## Supplementary Material

Additional file 1 **Pairwise relationship of the 43 animals.** The pairwise relationship of the 43 re-sequenced animals was extracted from the numerator relationship matrix. Different color indicates the extent of relationship. Click here for file

Additional file 2 **Ancestry of the re-sequenced animals.** The animals are ordered according to their birth year. Bold type indicates animals with re-sequenced sires. Click here for file

Additional file 3 Mapping statistics. Click here for file

Additional file 4 Counts of biallelic SNPs per animal. Click here for file

Additional file 5 **Prediction of functional effects.** The prediction of effects of non-synonymous variants (n=42,519) was performed by using *Polyphen2-2 v.2.2.2*[41]**.** Click here for file

Additional file 6 UMD3.1 coordinates of OMIA bovine causal variants. Click here for file

Additional file 7 References of articles cited in Additional file 6. Click here for file

Additional file 8 **Distribution of read lengths per sequenced animal.** For each animal, one library was constructed. Eight animals were sequenced in two or more different runs resulting in different of read lengths. Click here for file

Additional file 9 **Determination of the boundary between the pseudoautosomal and non-pseudoautosomal regions on the bovine X chromosome.** High-density genotypes (777,962 SNPs per animal) were available for 896 male animals of the Fleckvieh population. The chromosomal position of the SNPs was determined according to the University of Maryland assembly of the bovine genome (UMD3.1). Animals and SNPs with call rates < 90 percent were omitted. The proportion of heterozygous animals was calculated for 38,868 SNPs on the X chromosome. The shaded box highlights the pseudoautosomal region that was estimated to extend from base 137,000,000 to the distal chromosome end because of the increased heterozygosity in this stretch. The pseudoautosomal region was subsequently treated like an autosomal area. Click here for file

Additional file 10 **Newly annotated genes.** For 32 genes the genomic structure was predicted based on the University of Maryland UMD3.1 assembly of the bovine genome sequences [10] and the Dana-Farber Cancer Institute bovine gene index release 12.0 [42] by using GENOMETHREADER software tool [43]. The GENOMETHREADER output was viewed and edited using the Apollo sequence annotation editor [44]. Gene: gene symbol; Chr (strand): chromosome (orientation of gene); txStart: position transcription start; txEnd: position transcription end; cdsStart: position coding start; cdsEnd: position of coding end; exons: number of exons. Click here for file
