# Supplementary figures and images for: Assessment of the genomic variation in a cattle population by re-sequencing of key animals at low to medium coverage
Source: BMC Genomics. 2013 Jul 4;14:446. doi: 10.1186/1471-2164-14-446 (PMC3716689; doi:10.1186/1471-2164-14-446)

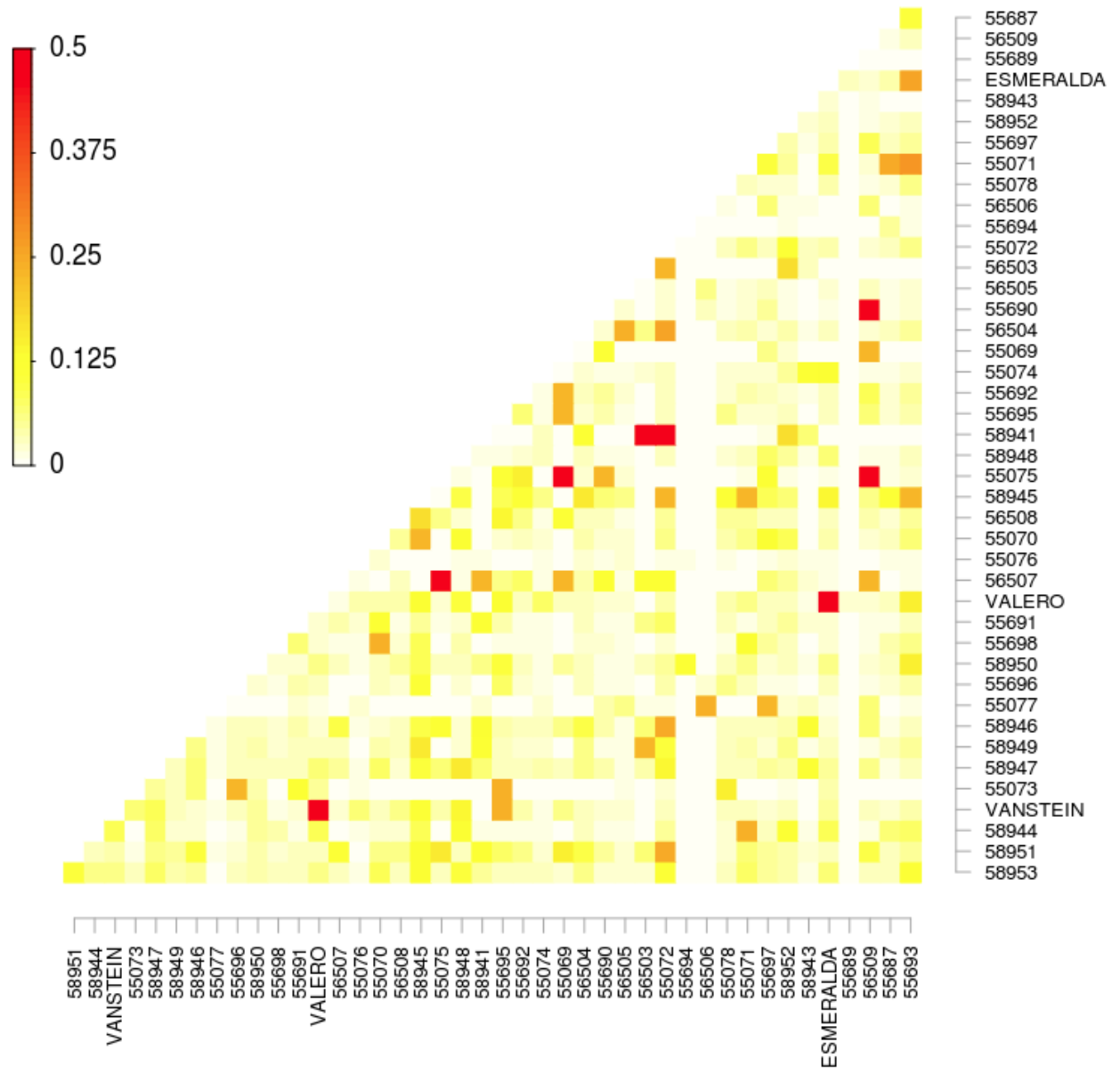

Supplement: Additional file 1 — Pairwise relationship of the 43 animals. The pairwise relationship of the 43 re-sequenced animals was extracted from the numerator relationship matrix. Different color indicates the extent of relationship. [file 1471-2164-14-446-S1.pdf]

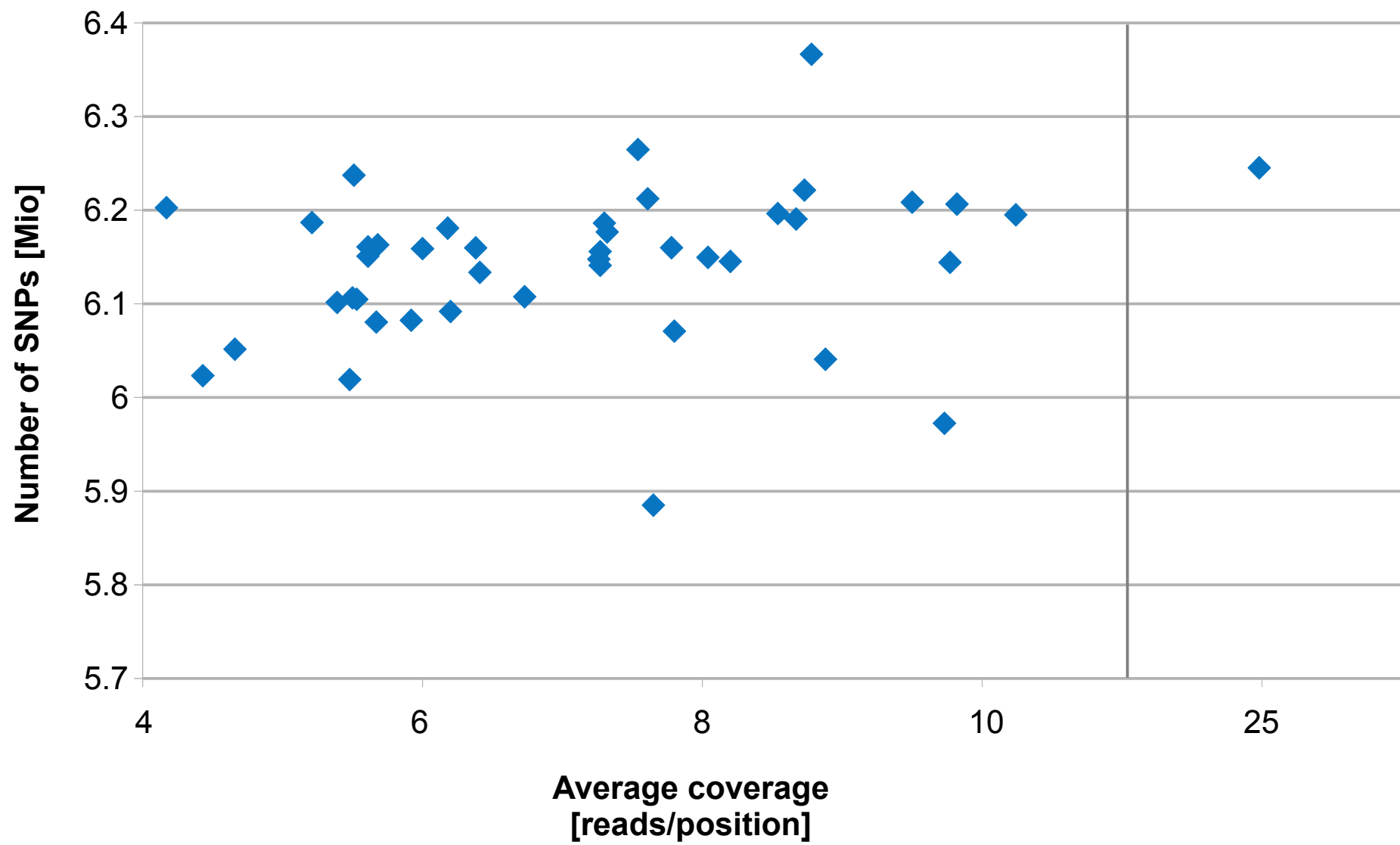

Supplement: Additional file 4 — Counts of biallelic SNPs per animal. [file 1471-2164-14-446-S4.pdf]

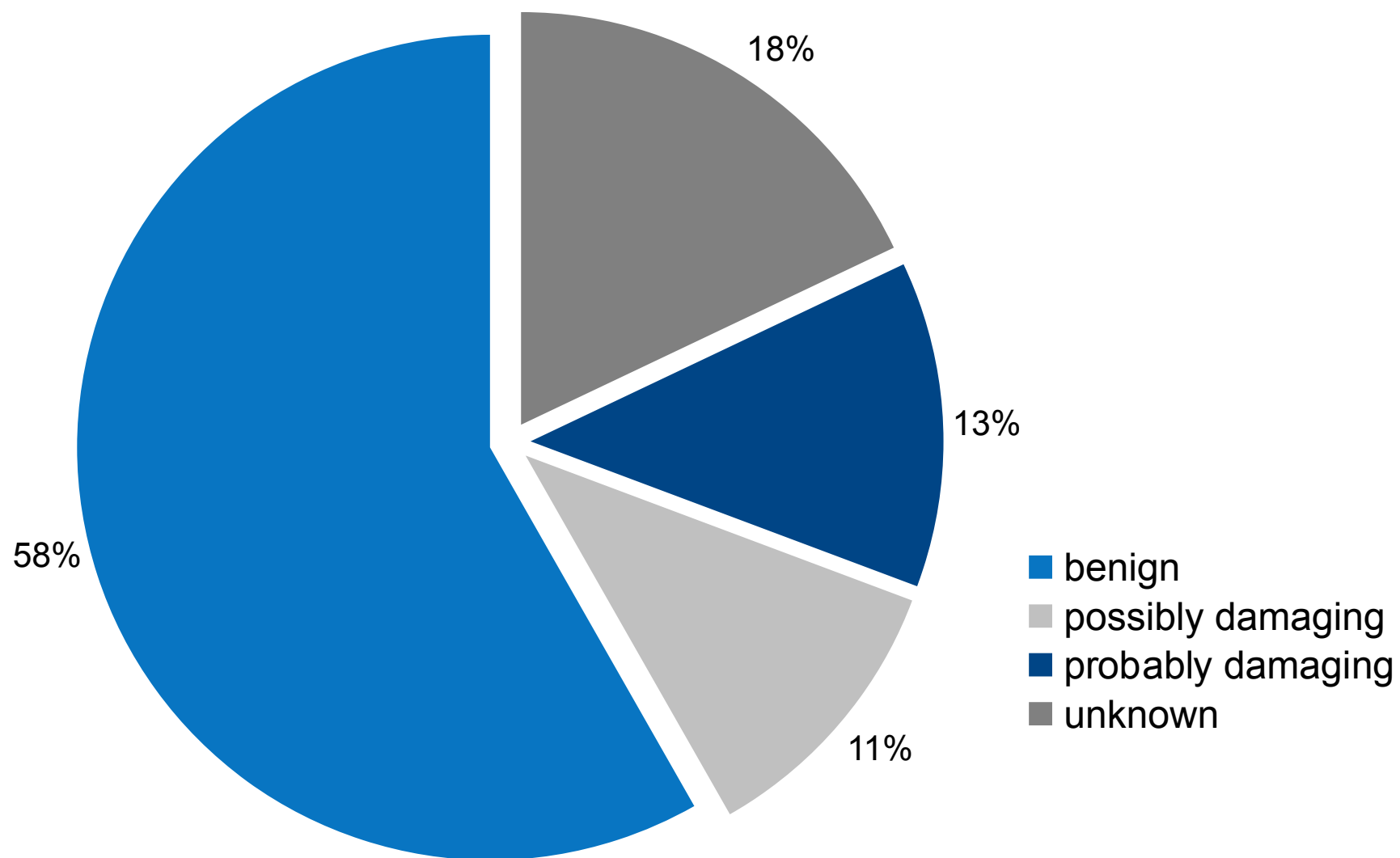

Supplement: Additional file 5 — Prediction of functional effects. The prediction of effects of non-synonymous variants (n=42,519) was performed by using Polyphen2-2 v.2.2.2[41]. [file 1471-2164-14-446-S5.pdf]

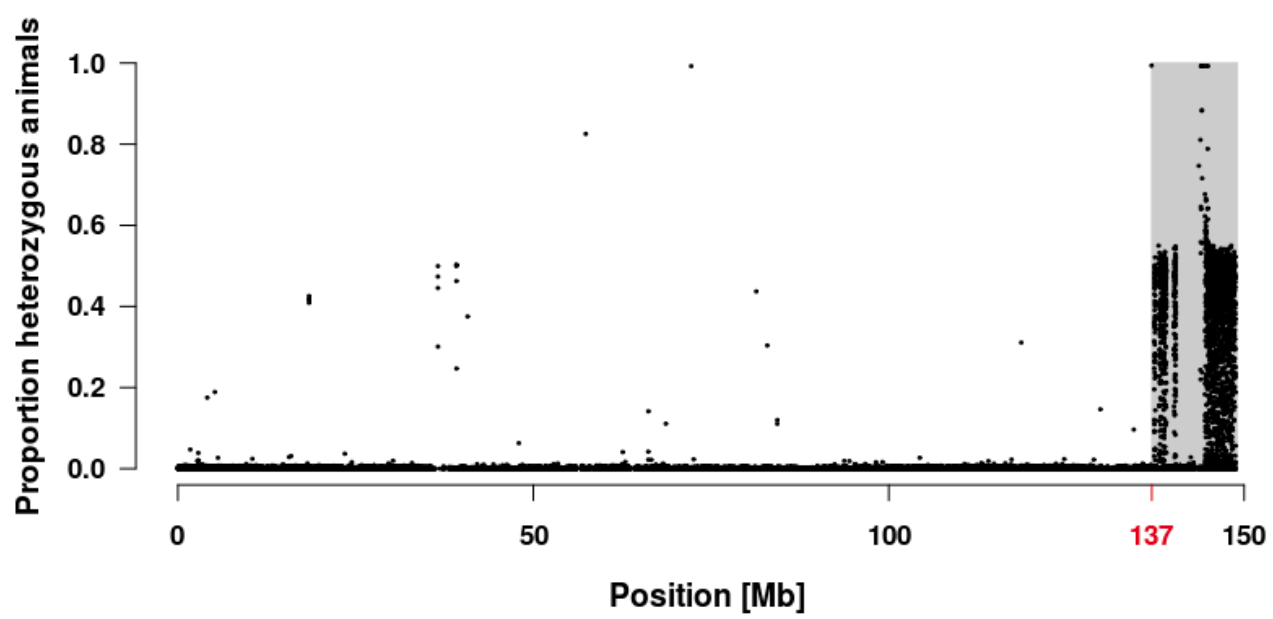

Supplement: Additional file 9 — Determination of the boundary between the pseudoautosomal and non-pseudoautosomal regions on the bovine X chromosome. High-density genotypes (777,962 SNPs per animal) were available for 896 male animals of the Fleckvieh population. The chromosomal position of the SNPs was determined according to the University of Maryland assembly of the bovine genome (UMD3.1). Animals and SNPs with call rates < 90 percent were omitted. The proportion of heterozygous animals was calculated for 38,868 SNPs on the X chromosome. The shaded box highlights the pseudoautosomal region that was estimated to extend from base 137,000,000 to the distal chromosome end because of the increased heterozygosity in this stretch. The pseudoautosomal region was subsequently treated like an autosomal area. [file 1471-2164-14-446-S9.pdf]
