# Supplementary material for: Assessment of the genomic variation in a cattle population by re-sequencing of key animals at low to medium coverage
Source: BMC Genomics. 2013 Jul 4;14:446. doi: 10.1186/1471-2164-14-446 (PMC3716689; doi:10.1186/1471-2164-14-446)
Supplement: Additional file 2 — Ancestry of the re-sequenced animals. The animals are ordered according to their birth year. Bold type indicates animals with re-sequenced sires. [file 1471-2164-14-446-S2.pdf]

Lab-ID	ISO-number	Name	Birth-year	Sire
58945	276000979317838	HAXL	1966	HAX
55069	756711620016730	REDAD	1973	JAMES-RED
55073	276000925265555	BALBO	1974	BALI
55071	276000929189864	ROMULUS	1975	ROXI
55070	276000929276244	STREIF	1978	STREITER
58941	276000809706945	HORROR	1979	HORNUNG
55077	276000926428551	POSTILLION	1981	POSCO
**55075**	**276000928504510**	**RENNER**	**1981**	**REDAD**
56506	276000920747734	PROPELLER	1981	POSCO
55694	276000926189400	DIRTECK	1983	DIRIGENT
58943	276000804179455	HODSCHA	1983	HOLB
55689	276007600026785	HOLZER PP	1985	HOLWIG
**55072**	**276000912851233**	**HORWEIN**	**1986**	**HORROR**
**56503**	**276000915581022**	**HORB**	**1986**	**HORROR**
58944	276000912851741	MORWEL	1986	MORELLO
55696	276000919587582	HALLING	1988	HALL
55695	276000911825633	RALBO	1988	RADI
55693	276000912971290	ROMEN	1988	ROM
55692	276000913008210	REXON	1989	REX
**56509**	**276000915732780**	**RENGER**	**1989**	**RENNER**
**56507**	**276000919598352**	**REPORT**	**1989**	**RENNER**
55687	276000910865689	ROMANEK	1989	ROMANUS
55074	276000910915308	EGOL	1991	EGEL
56508	276000919253926	UTNACH	1991	UTERINO
55698	276000910950070	SPORT	1992	STRESS
55076	276000913892370	SAMURAI	1992	STEG
55078	276000910794565	GEBAL	1992	GEHA
55691	276000911474933	BOSS	1993	BAMBI
56505	276000915040032	HUMLANG	1994	HUMBERG
55697	276000913325437	POLDI	1996	POSTNER
**55690**	**276000918174246**	**REGIO**	**1996**	**RENGER**
56504	276000918912889	HUMID	1996	HUMBERG
58951	276000933663105	WEINOLD	1999	WEINOX
58946	276000932739095	WATERBERG	1999	WINZER
58948	276000931098195	MARTL DE	1999	GS MALF
58949	276000933038755	ZAHNER	2000	ZAHN
VANSTEIN	276000934586859	VANSTEIN	2000	RANDY
58947	276000935684041	MANDELA	2001	MALEFIZ
58950	276000938263111	DAGO	2004	GS DIONIS
ESMERALDA	276000938065319	ESMERALDA P	2004	ROMELLO
58952	276000940823906	MORGENGRAU	2006	MORIS
**VALERO**	**276000941364903**	**VALERO PS**	**2007**	**VANSTEIN**
58953	276000943707915		2009	HEXER
