# Supplementary material for: Assessment of the genomic variation in a cattle population by re-sequencing of key animals at low to medium coverage
Source: BMC Genomics. 2013 Jul 4;14:446. doi: 10.1186/1471-2164-14-446 (PMC3716689; doi:10.1186/1471-2164-14-446)
Supplement: Additional file 3 — Mapping statistics. [file 1471-2164-14-446-S3.pdf]

Animal id	Total reads	Duplicates [a]	Mapped reads [b]	Net mapped (%) [c]	Net bases [d]	Net average coverage [e]
55069	163482913	5.17%	96.43%	149489938 (91.44%)	15.10GB	5.68
55070	248209072	6.50%	97.90%	227199055 (91.54%)	22.72GB	8.54
55071	275347611	6.38%	98.04%	252713383 (91.78%)	25.27GB	9.50
55072	218095293	5.92%	97.72%	200490494 (91.93%)	20.05GB	7.54
55073	173087784	5.26%	96.37%	158034806 (91.30%)	15.96GB	6.00
55074	156705901	5.86%	96.28%	142031377 (90.64%)	14.35GB	5.39
55075	257059233	7.06%	97.79%	233629804 (90.89%)	23.36GB	8.78
55076	295394459	4.65%	97.04%	273327445 (92.53%)	27.24GB	10.24
55077	251461184	5.40%	96.89%	230495110 (91.66%)	23.05GB	8.67
55078	284445379	5.65%	96.90%	260051305 (91.42%)	25.98GB	9.77
55687	162759413	7.00%	96.14%	145527098 (89.41%)	14.70GB	5.53
55689	287537748	7.35%	97.72%	260332777 (90.54%)	25.87GB	9.73
55690	160290585	4.83%	98.05%	149570539 (93.31%)	14.66GB	5.51
55691	123602395	6.43%	95.93%	110944671 (89.76%)	11.09GB	4.17
55692	138843685	6.86%	95.81%	123887746 (89.23%)	12.39GB	4.66
55693	131466898	7.41%	96.80%	117828683 (89.63%)	11.78GB	4.43
55694	150863976	5.59%	97.29%	138578560 (91.86%)	13.86GB	5.21
55695	261211185	8.41%	96.46%	230777234 (88.35%)	23.22GB	8.73
55696	174223713	8.02%	97.64%	156464263 (89.81%)	15.74GB	5.92
55697	245108260	8.48%	97.18%	217996621 (88.94%)	21.80GB	8.20
55698	270847013	8.83%	95.63%	236135825 (87.18%)	23.61GB	8.88
56503	181507121	4.61%	97.99%	169656055 (93.47%)	16.97GB	6.38
56504	160842497	4.16%	97.84%	150815249 (93.77%)	15.08GB	5.67
56505	227151631	4.29%	98.35%	213806924 (94.13%)	21.38GB	8.04
56506	190028977	4.09%	98.18%	178943367 (94.17%)	17.89GB	6.73
56507	208459066	4.91%	98.23%	194715327 (93.41%)	19.47GB	7.32
56508	207068014	4.59%	98.23%	194066215 (93.72%)	19.41GB	7.30
56509	176164262	4.71%	97.99%	164482114 (93.37%)	16.45GB	6.18
58941	161865174	5.16%	96.24%	147729603 (91.27%)	14.92GB	5.61
58943	226335891	4.35%	95.54%	206832039 (91.38%)	20.68GB	7.78
58944	216683526	3.59%	96.85%	202333113 (93.38%)	20.23GB	7.61
58945	207869816	4.05%	96.97%	193404741 (93.04%)	19.34GB	7.27
58946	208384577	4.45%	97.03%	193209439 (92.72%)	19.32GB	7.26
58947	182548630	3.72%	97.06%	170592510 (93.45%)	17.06GB	6.41
58948	160719456	4.32%	97.13%	149353352 (92.93%)	14.94GB	5.61
58949	160661919	6.47%	96.48%	144980005 (90.24%)	14.64GB	5.50
58950	212036734	5.66%	96.65%	193340167 (91.18%)	19.33GB	7.27
58951	160626127	6.89%	96.41%	144198385 (89.77%)	14.56GB	5.48
58952	178349820	4.57%	96.93%	164966017 (92.50%)	16.50GB	6.20
58953	225268008	5.15%	97.06%	207389836 (92.06%)	20.74GB	7.80
esmeralda	974913554	15.55%	96.92%	797934242 (81.85%)	66.44GB	24.98
valero	332400410	12.79%	90.06%	261080902 (78.54%)	26.11GB	9.82
vanstein	686227977	13.17%	94.87%	565303870 (82.38%)	20.35GB	7.65

[a] PCR-duplicates arising during the amplification step of the library preparation and marked with MarkDuplicates option of the Picard tools [1]

[b] Proportion of reads that have been mapped successfully to the UMD3.1 assembly [2] with bwa [3]

[c] Number of mapped reads excluding duplicates

[d] Number of net mapped reads multiplied by read lengths (see Additional file 2)

[e] Number of net bases divided by the length of the genome (~2.67 Gb)

References

1. **http://picard.sourceforge.net**. .

2. Zimin AV, Delcher AL, Florea L, Kelley DR, Schatz MC, Puiu D, Hanrahan F, Pertea G, Van Tassell CP, Sonstegard TS, Marçais G, Roberts M, Subramanian P, Yorke JA, Salzberg SL: **A whole-genome assembly of the domestic cow, Bos taurus**. *Genome Biol* 2009, **10**:R42.

3. Li H, Durbin R: **Fast and accurate short read alignment with Burrows–Wheeler transform**. *Bioinformatics* 2009, **25**:1754 –1760.
