# Supplementary material for: Assessment of the genomic variation in a cattle population by re-sequencing of key animals at low to medium coverage
Source: BMC Genomics. 2013 Jul 4;14:446. doi: 10.1186/1471-2164-14-446 (PMC3716689; doi:10.1186/1471-2164-14-446)
Supplement: Additional file 7 — References of articles cited in Additional file 6. [file 1471-2164-14-446-S7.pdf]

1. Charlier C, Agerholm JS, Coppieters W, Karlskov-Mortensen P, Li W, de Jong G, Fasquelle C, Karim L, Cirera S, Cambisano N, Ahariz N, Mullaart E, Georges M, Fredholm M: **A deletion in the bovine FANCI gene compromises fertility by causing fetal death and brachyspina**. *PLoS ONE* 2012, **7**:e43085.

2. Cavanagh JAL, Tammen I, Windsor PA, Bateman JF, Savarirayan R, Nicholas FW, Raadsma HW: **Bulldog dwarfism in Dexter cattle is caused by mutations in ACAN**. *Mamm Genome* 2007, **18**:808-814.

3. Colige A, Sieron AL, Li SW, Schwarze U, Petty E, Wertelecki W, Wilcox W, Krakow D, Cohn DH, Reardon W, Byers PH, Lapière CM, Prockop DJ, Nusgens BV: **Human Ehlers-Danlos syndrome type VII C and bovine dermatosparaxis are caused by mutations in the procollagen I N-proteinase gene**. *Am J Hum Genet* 1999, **65**:308-317.

4. Girardot M, Guibert S, Laforet M-P, Gallard Y, Larroque H, Oulmouden A: **The insertion of a full-length Bos taurus LINE element is responsible for a transcriptional deregulation of the Normande Agouti gene**. *Pigment Cell Res* 2006, **19**:346-355.

5. Dennis JA, Healy PJ: **Definition of the mutation responsible for maple syrup urine disease in Poll Shorthorns and genotyping Poll Shorthorns and Poll Herefords for maple syrup urine disease alleles**. *Res Vet Sci* 1999, **67**:1-6.

6. Drögemüller C, Drögemüller M, Leeb T, Mascarello F, Testoni S, Rossi M, Gentile A, Damiani E, Sacchetto R: **Identification of a missense mutation in the bovine ATP2A1 gene in congenital pseudomyotonia of Chianina cattle: an animal model of human Brody disease**. *Genomics* 2008, **92**:474-477.

7. Zhang B, Healy PJ, Zhao Y, Crabb DW, Harris RA: **Premature translation termination of the pre-E1 alpha subunit of the branched chain alpha-ketoacid dehydrogenase as a cause of maple syrup urine disease in Polled Hereford calves**. *J Biol Chem* 1990, **265**:2425-2427.

8. Dennis JA, Healy PJ, Beaudet AL, O'Brien WE: **Molecular definition of bovine argininosuccinate synthetase deficiency**. *Proc Natl Acad Sci USA* 1989, **86**:7947-7951.

9. Berry SD, Davis SR, Beattie EM, Thomas NL, Burrett AK, Ward HE, Stanfield AM, Biswas M, Ankersmit-Udy AE, Oxley PE, Barnett JL, Pearson JF, van der Does Y, Macgibbon AHK, Spelman RJ, Lehnert K, Snell RG: **Mutation in bovine beta-carotene oxygenase 2 affects milk color**. *Genetics* 2009, **182**:923-926.

10. Kraner S, Sieb J, Thompson P, Steinlein O: **Congenital myasthenia in Brahman calves caused by homozygosity for a CHRNE truncating mutation**. *neurogenetics* 2002, **4**:87-91.

11. Thompson PN, Steinlein OK, Harper CK, Kraner S, Sieb JP, Guthrie AJ: **Congenital myasthenic syndrome of Brahman cattle in South Africa**. *Vet Rec* 2003, **153**:779-781.

12. Hirano T, Kobayashi N, Itoh T, Takasuga A, Nakamaru T, Hirotsune S, Sugimoto Y: **Null Mutation of PCLN-1/Claudin-16 Results in Bovine Chronic Interstitial Nephritis**. *Genome Res* 2000, **10**:659-663.

13. Hirano T, Hirotsune S, Sasaki S, Kikuchi T, Sugimoto Y: **A new deletion mutation in bovine Claudin-16 (CL-16) deficiency and diagnosis**. *Animal Genetics* 2002, **33**:118–122.

14. Houweling PJ, Cavanagh JAL, Palmer DN, Frugier T, Mitchell NL, Windsor PA, Raadsma HW, Tammen I: **Neuronal ceroid lipofuscinosis in Devon cattle is caused by a single base duplication**

(c.662dupG) in the bovine CLN5 gene. *Biochimica et Biophysica Acta (BBA) - Molecular Basis of Disease* 2006, **1762**:890-897.

15. Drögemüller C, Distl O, Leeb T: **Partial Deletion of the Bovine ED1 Gene Causes Anhidrotic Ectodermal Dysplasia in Cattle**. *Genome Res* 2001, **11**:1699-1705.

16. Drögemüller C, Peters M, Pohlenz J, Distl O, Leeb T: **A single point mutation within the ED1 gene disrupts correct splicing at two different splice sites and leads to anhidrotic ectodermal dysplasia in cattle**. *Journal of Molecular Medicine* 2002, **80**:319-323.

17. Tajima M, Miyake S, Takehana K, Kobayashi A, Yamato O, Maede Y: **Gene Defect of Dermatan Sulfate Proteoglycan of Cattle Affected With a Variant Form of Ehlers-Danlos Syndrome**. *Journal of Veterinary Internal Medicine* 1999, **13**:202–205.

18. Takeda H, Takami M, Oguni T, Tsuji T, Yoneda K, Sato H, Ihara N, Itoh T, Kata SR, Mishina Y, Womack JE, Moritomo Y, Sugimoto Y, Kunieda T: **Positional cloning of the gene LIMBIN responsible for bovine chondrodysplastic dwarfism**. *Proc Natl Acad Sci USA* 2002, **99**:10549-10554.

19. Marron BM, Robinson JL, Gentry PA, Beever JE: **Identification of a mutation associated with factor XI deficiency in Holstein cattle**. *Animal Genetics* 2004, **35**:454–456.

20. Kunieda M, Tsuji T, Abbasi AR, Khalaj M, Ikeda M, Miyadera K, Ogawa H, Kunieda T: **An insertion mutation of the bovine Fii gene is responsible for factor XI deficiency in Japanese black cattle**. *Mamm Genome* 2005, **16**:383-389.

21. Khalaj M, Abbasi AR, Shimojo K, Moritomo Y, Yoneda K, Kunieda T: **A missense mutation (p.Leu2153His) of the factor VIII gene causes cattle haemophilia A**. *Anim Genet* 2009, **40**:763-765.

22. Singleton AC, Mitchell AL, Byers PH, Potter KA, Pace JM: **Bovine model of Marfan syndrome results from an amino acid change (c.3598G>A, p.E1200K) in a calcium-binding epidermal growth factor-like domain of fibrillin-1**. *Human Mutation* 2005, **25**:348–352.

23. Hirano T, Matsuhashi T, Kobayashi N, Watanabe T, Sugimoto Y: **Identification of an FBN1 mutation in bovine Marfan syndrome-like disease**. *Animal Genetics* 2012, **43**:11–17.

24. Jenkins MM, LeBoeuf RD, Ruth GR, Bloomer JR: **A novel stop codon mutation (X417L) of the ferrochelatase gene in bovine protoporphyria, a natural animal model of the human disease**. *Biochimica et Biophysica Acta (BBA) - Molecular Basis of Disease* 1998, **1408**:18-24.

25. Lundén A, Marklund S, Gustafsson V, Andersson L: **A Nonsense Mutation in the FMO3 Gene Underlies Fishy Off-Flavor in Cow's Milk**. *Genome Res* 2002, **12**:1885-1888.

26. Dennis JA, Moran C, Healy PJ: **The bovine α-glucosidase gene: coding region, genomic structure, and mutations that cause bovine generalized glycogenosis**. *Mammalian Genome* 2000, **11**:206-212.

27. McCormack BL, Chase Jr. CC, Olson TA, Elsasser TH, Hammond AC, Welsh Jr. TH, Jiang H, Randel RD, Okamura CA, Lucy MC: **A miniature condition in Brahman cattle is associated with a single nucleotide mutation within the growth hormone gene**. *Domestic Animal Endocrinology* 2009, **37**:104-111.

28. Pierce KD, Handford CA, Morris R, Vafa B, Dennis JA, Healy PJ, Schofield PR: **A Nonsense**

Mutation in the α1 Subunit of the Inhibitory Glycine Receptor Associated with Bovine Myoclonus. *Molecular and Cellular Neuroscience* 2001, **17**:354-363.

29. Shuster DE, Kehrli ME Jr, Ackermann MR, Gilbert RO: **Identification and prevalence of a genetic defect that causes leukocyte adhesion deficiency in Holstein cattle**. *Proc Natl Acad Sci USA* 1992, **89**:9225-9229.

30. Krebs S, Medugorac I, Röther S, Strässer K, Förster M: **A missense mutation in the 3-ketodihydrosphingosine reductase FVT1 as candidate causal mutation for bovine spinal muscular atrophy**. *Proc Natl Acad Sci USA* 2007, **104**:6746-6751.

31. Seitz JJ, Schmutz SM, Thue TD, Buchanan FC: **A missense mutation in the bovine MGF gene is associated with the roan phenotype in Belgian Blue and Shorthorn cattle**. *Mamm Genome* 1999, **10**:710-712.

32. Ford CA, Stanfield AM, Spelman RJ, Smits B, Ankersmidt-Udy AEL, Cottier K, Holloway H, Walden A, Al-Wahb M, Bohm E, Snell RG, Sutherland GT: **A mutation in bovine keratin 5 causing epidermolysis bullosa simplex, transmitted by a mosaic sire**. *J Invest Dermatol* 2005, **124**:1170-1176.

33. Duchesne A, Gautier M, Chadi S, Grohs C, Floriot S, Gallard Y, Caste G, Ducos A, Eggen A: **Identification of a doublet missense substitution in the bovine LRP4 gene as a candidate causal mutation for syndactyly in Holstein cattle**. *Genomics* 2006, **88**:610-621.

34. Johnson EB, Steffen DJ, Lynch KW, Herz J: **Defective splicing of Megf7/Lrp4, a regulator of distal limb development, in autosomal recessive mulefoot disease**. *Genomics* 2006, **88**:600-609.

35. Drögemüller C, Leeb T, Harlizius B, Tammen I, Distl O, Höltershinken M, Gentile A, Duchesne A, Eggen A: **Congenital syndactyly in cattle: four novel mutations in the low density lipoprotein receptor-related protein 4 gene (LRP4)**. *BMC Genet* 2007, **8**:5.

36. Kunieda T, Nakagiri M, Takami M, Ide H, Ogawa H: **Cloning of bovine LYST gene and identification of a missense mutation associated with Chediak-Higashi syndrome of cattle**. *Mamm Genome* 1999, **10**:1146-1149.

37. Tollersrud OK, Berg T, Healy P, Evjen G, Ramachandran U, Nilssen Ø: **Purification of Bovine Lysosomal α-Mannosidase, Characterization of its Gene and Determination of two Mutations that Cause α-Mannosidosis**. *European Journal of Biochemistry* 1997, **246**:410–419.

38. Leipprandt JR, Chen H, Horvath JE, Qiao XT, Jones MZ, Friderici KH: **Identification of a bovine beta-mannosidosis mutation and detection of two beta-mannosidase pseudogenes**. *Mamm Genome* 1999, **10**:1137-1141.

39. Klungland H, Våge DI, Gomez-Raya L, Adalsteinsson S, Lien S: **The role of melanocyte-stimulating hormone (MSH) receptor in bovine coat color determination**. *Mamm Genome* 1995, **6**:636-639.

40. Drögemüller C, Reichart U, Seuberlich T, Oevermann A, Baumgartner M, Kühni Boghenbor K, Stoffel MH, Syring C, Meylan M, Müller S, Müller M, Gredler B, Sölkner J, Leeb T: **An unusual splice defect in the mitofusin 2 gene (MFN2) is associated with degenerative axonopathy in Tyrolean Grey cattle**. *PLoS ONE* 2011, **6**:e18931.

41. Fontanesi L, Scotti E, Russo V: **Haplotype variability in the bovine MITF gene and association with piebaldism in Holstein and Simmental cattle breeds**. *Anim Genet* 2012, **43**:250-

256.

42. Philipp U, Lupp B, Mömke S, Stein V, Tipold A, Eule JC, Rehage J, Distl O: **A MITF mutation associated with a dominant white phenotype and bilateral deafness in German Fleckvieh cattle**. *PLoS ONE* 2011, **6**:e28857.

43. Buitkamp J, Semmer J, Götz K-U: **Arachnomelia syndrome in Simmental cattle is caused by a homozygous 2-bp deletion in the molybdenum cofactor synthesis step 1 gene (MOCS1)**. *BMC Genet* 2011, **12**:11.

44. Fasquelle C, Sartelet A, Li W, Dive M, Tamma N, Michaux C, Druet T, Huijbers IJ, Isacke CM, Coppieters W, Georges M, Charlier C: **Balancing selection of a frame-shift mutation in the MRC2 gene accounts for the outbreak of the Crooked Tail Syndrome in Belgian Blue Cattle**. *PLoS Genet* 2009, **5**:e1000666.

45. Sartelet A, Klingbeil P, Franklin CK, Fasquelle C, Géron S, Isacke CM, Georges M, Charlier C: **Allelic heterogeneity of Crooked Tail Syndrome: result of balancing selection?** *Anim Genet* 2012, **43**:604-607.

46. Grobet L, Martin LJ, Poncelet D, Pirottin D, Brouwers B, Riquet J, Schoeberlein A, Dunner S, Ménissier F, Massabanda J, Fries R, Hanset R, Georges M: **A deletion in the bovine myostatin gene causes the double-muscled phenotype in cattle**. *Nat Genet* 1997, **17**:71-74.

47. Kambadur R, Sharma M, Smith TP, Bass JJ: **Mutations in myostatin (GDF8) in double-muscled Belgian Blue and Piedmontese cattle**. *Genome Res* 1997, **7**:910-916.

48. Grobet L, Poncelet D, Royo LJ, Brouwers B, Pirottin D, Michaux C, Ménissier F, Zanotti M, Dunner S, Georges M: **Molecular definition of an allelic series of mutations disrupting the myostatin function and causing double-muscling in cattle**. *Mamm Genome* 1998, **9**:210-213.

49. Karim L, Coppieters W, Grobet L, Valentini A, Georges M: **Convenient genotyping of six myostatin mutations causing double-muscling in cattle using a multiplex oligonucleotide ligation assay**. *Anim Genet* 2000, **31**:396-399.

50. Karageorgos L, Hill B, Bawden MJ, Hopwood JJ: **Bovine mucopolysaccharidosis type IIIB**. *J Inherit Metab Dis* 2007, **30**:358-364.

51. Owczarek-Lipska M, Plattet P, Zipperle L, Drögemüller C, Posthaus H, Dolf G, Braunschweig MH: **A nonsense mutation in the optic atrophy 3 gene (OPA3) causes dilated cardiomyopathy in Red Holstein cattle**. *Genomics* 2011, **97**:51-57.

52. Braunschweig MH, Leeb T: **Aberrant low expression level of bovine beta-lactoglobulin is associated with a C to A transversion in the BLG promoter region**. *J Dairy Sci* 2006, **89**:4414-4419.

53. Kühn C, Weikard R: **An investigation into the genetic background of coat colour dilution in a Charolais x German Holstein F2 resource population**. *Anim Genet* 2007, **38**:109-113.

54. Jolly RD, Wills JL, Kenny JE, Cahill JI, Howe L: **Coat-colour dilution and hypotrichosis in Hereford crossbred calves**. *N Z Vet J* 2008, **56**:74-77.

55. Schmutz SM, Dreger DL: **Interaction of MC1R and PMEL alleles on solid coat colors in Highland cattle**. *Animal genetics* 2013, **44(1)**:9-13.

56. Simpson MA, Cook RW, Solanki P, Patton MA, Dennis JA, Crosby AH: **A mutation in NFkappaB interacting protein 1 causes cardiomyopathy and woolly haircoat syndrome of Poll Hereford cattle**. *Anim Genet* 2009, **40**:42-46.

57. Koltes JE, Mishra BP, Kumar D, Kataria RS, Totir LR, Fernando RL, Cobbold R, Steffen D, Coppieters W, Georges M, Reecy JM: **A nonsense mutation in cGMP-dependent type II protein kinase (PRKG2) causes dwarfism in American Angus cattle**. *Proc Natl Acad Sci USA* 2009, **106**:19250-19255.

58. Tsujino S, Shanske S, Valberg SJ, Cardinet GH 3rd, Smith BP, DiMauro S: **Cloning of bovine muscle glycogen phosphorylase cDNA and identification of a mutation in cattle with myophosphorylase deficiency, an animal model for McArdle's disease**. *Neuromuscul Disord* 1996, **6**:19-26.

59. Johnstone AC, McSporran KD, Kenny JE, Anderson IL, Macpherson GR, Jolly RD: **Myophosphorylase deficiency (glycogen storage disease Type V) in a herd of Charolais cattle in New Zealand: confirmation by PCR-RFLP testing**. *N Z Vet J* 2004, **52**:404-408.

60. Boudreaux MK, Schmutz SM, French PS: **Calcium diacylglycerol guanine nucleotide exchange factor I (CalDAG-GEFI) gene mutations in a thrombopathic Simmental calf**. *Vet Pathol* 2007, **44**:932-935.

61. Sartelet A, Druet T, Michaux C, Fasquelle C, Géron S, Tamma N, Zhang Z, Coppieters W, Georges M, Charlier C: **A Splice Site Variant in the Bovine RNF11 Gene Compromises Growth and Regulation of the Inflammatory Response**. *PLoS Genet* 2012, **8**:e1002581.

62. Thomsen B, Horn P, Panitz F, Bendixen E, Petersen AH, Holm L-E, Nielsen VH, Agerholm JS, Arnbjerg J, Bendixen C: **A missense mutation in the bovine SLC35A3 gene, encoding a UDP-N-acetylglucosamine transporter, causes complex vertebral malformation**. *Genome Res* 2006, **16**:97-105.

63. Yuzbasiyan-Gurkan V, Bartlett E: **Identification of a unique splice site variant in SLC39A4 in bovine hereditary zinc deficiency, lethal trait A46: An animal model of acrodermatitis enteropathica**. *Genomics* 2006, **88**:521-526.

64. Inaba M, Yawata A, Koshino I, Sato K, Takeuchi M, Takakuwa Y, Manno S, Yawata Y, Kanzaki A, Sakai J, Ban A, Ono K, Maede Y: **Defective anion transport and marked spherocytosis with membrane instability caused by hereditary total deficiency of red cell band 3 in cattle due to a nonsense mutation**. *J Clin Invest* 1996, **97**:1804-1817.

65. Meyers SN, McDaneld TG, Swist SL, Marron BM, Steffen DJ, O'Toole D, O'Connell JR, Beever JE, Sonstegard TS, Smith TPL: **A deletion mutation in bovine SLC4A2 is associated with osteopetrosis in Red Angus cattle**. *BMC Genomics* 2010, **11**:337.

66. Charlier C, Coppieters W, Rollin F, Desmecht D, Agerholm JS, Cambisano N, Carta E, Dardano S, Dive M, Fasquelle C, Frennet J-C, Hanset R, Hubin X, Jorgensen C, Karim L, Kent M, Harvey K, Pearce BR, Simon P, Tama N, Nie H, Vandeputte S, Lien S, Longeri M, Fredholm M, Harvey RJ, Georges M: **Highly effective SNP-based association mapping and management of recessive defects in livestock**. *Nat Genet* 2008, **40**:449-454.

67. Thomsen B, Nissen PH, Agerholm JS, Bendixen C: **Congenital bovine spinal dysmyelination is caused by a missense mutation in the SPAST gene**. *Neurogenetics* 2010, **11**:175-183.

68. Drögemüller C, Tetens J, Sigurdsson S, Gentile A, Testoni S, Lindblad-Toh K, Leeb T:

Identification of the bovine Arachnomelia mutation by massively parallel sequencing implicates sulfite oxidase (SUOX) in bone development. *PLoS Genet* 2010, **6**.

69. Ricketts MH, Simons MJ, Parma J, Mercken L, Dong Q, Vassart G: **A nonsense mutation causes hereditary goitre in the Afrikander cattle and unmasks alternative splicing of thyroglobulin transcripts**. *Proc Natl Acad Sci USA* 1987, **84**:3181-3184.

70. Capitan A, Grohs C, Weiss B, Rossignol M-N, Reversé P, Eggen A: **A Newly Described Bovine Type 2 Scurs Syndrome Segregates with a Frame-Shift Mutation in TWIST1**. *PLoS ONE* 2011, **6**:e22242.

71. Schmutz SM, Berryere TG, Ciobanu DC, Mileham AJ, Schmidtz BH, Fredholm M: **A form of albinism in cattle is caused by a tyrosinase frameshift mutation**. *Mamm Genome* 2004, **15**:62-67.

72. Berryere TG, Schmutz SM, Schimpf RJ, Cowan CM, Potter J: **TYRP1 is associated with dun coat colour in Dexter cattle or how now brown cow?** *Anim Genet* 2003, **34**:169-175.

73. Schwenger B, Schöber S, Simon D: **DUMPS cattle carry a point mutation in the uridine monophosphate synthase gene**. *Genomics* 1993, **16**:241-244.

74. Abbasi AR, Khalaj M, Tsuji T, Tanahara M, Uchida K, Sugimoto Y, Kunieda T: **A mutation of the WFDC1 gene is responsible for multiple ocular defects in cattle**. *Genomics* 2009, **94**:55-62.
