# Supplementary material for: Assessment of the genomic variation in a cattle population by re-sequencing of key animals at low to medium coverage
Source: BMC Genomics. 2013 Jul 4;14:446. doi: 10.1186/1471-2164-14-446 (PMC3716689; doi:10.1186/1471-2164-14-446)
Supplement: Additional file 8 — Distribution of read lengths per sequenced animal. For each animal, one library was constructed. Eight animals were sequenced in two or more different runs resulting in different of read lengths. [file 1471-2164-14-446-S8.pdf]

Id	36 bp	76 bp	96 bp	100 bp	101 bp
55069	-	-	-	-	100.00%
55070	-	-	-	100.00%	-
55071	-	-	-	100.00%	-
55072	-	-	-	100.00%	-
55073	-	-	-	-	100.00%
55074	-	-	-	-	100.00%
55075	-	-	-	100.00%	-
55076	-	-	22.37%	22.37%	55.26%
55077	-	-	16.5%	16.5%	67.00%
55078	-	-	17.97%	17.98%	64.05%
55687	-	-	-	-	100.00%
55689	-	-	15.69%	84.31%	-
55690	-	-	49.99%	50.01%	-
55691	-	-	-	100.00%	-
55692	-	-	-	100.00%	-
55693	-	-	-	100.00%	-
55694	-	-	-	100.00%	-
55695	-	-	-	36.54%	63.46%
55696	-	-	-	37.3%	62.7%
55697	-	-	-	100.00%	-
55698	-	-	-	100.00%	-
56503	-	-	-	100.00%	-
56504	-	-	-	100.00%	-
56505	-	-	-	100.00%	-
56506	-	-	-	100.00%	-
56507	-	-	-	100.00%	-
56508	-	-	-	100.00%	-
56509	-	-	-	100.00%	-
58941	-	-	-	-	100.00%
58943	-	-	-	100.00%	-
58944	-	-	-	100.00%	-
58945	-	-	-	100.00%	-
58946	-	-	-	100.00%	-
58947	-	-	-	100.00%	-
58948	-	-	-	100.00%	-
58949	-	-	-	-	100.00%
58950	-	-	-	100.00%	-
58951	-	-	-	-	100.00%
58952	-	-	-	100.00%	-
58953	-	-	-	100.00%	-
esmeralda	-	69.75%	-	30.25%	-
vanstein	100.00%	-	-	-	-
valero	-	-	-	100.00%	-
