# Supplementary material for: Assessment of the genomic variation in a cattle population by re-sequencing of key animals at low to medium coverage
Source: BMC Genomics. 2013 Jul 4;14:446. doi: 10.1186/1471-2164-14-446 (PMC3716689; doi:10.1186/1471-2164-14-446)
Supplement: Additional file 10 — Newly annotated genes. For 32 genes the genomic structure was predicted based on the University of Maryland UMD3.1 assembly of the bovine genome sequences [10] and the Dana-Farber Cancer Institute bovine gene index release 12.0 [42] by using GENOMETHREADER software tool [43]. The GENOMETHREADER output was viewed and edited using the Apollo sequence annotation editor [44]. Gene: gene symbol; Chr (strand): chromosome (orientation of gene); txStart: position transcription start; txEnd: position transcription end; cdsStart: position coding start; cdsEnd: position of coding end; exons: number of exons. [file 1471-2164-14-446-S10.pdf]

For thirty-two genes listed below the genomic structure was predicted based on the University of Maryland UMD3.1 assembly of the bovine genome sequences [1] and the Dana-Farber Cancer Institute bovine gene index release 12.0 [2] by using GENOMETHREADER software tool [3]. The GENOMETHREADER output was viewed and edited using the Apollo sequence annotation editor [4].

1. Zimin AV, Delcher AL, Florea L, Kelley DR, Schatz MC, Puiu D, Hanrahan F, Pertea G, Van Tassell CP, Sonstegard TS, Marçais G, Roberts M, Subramanian P, Yorke JA, Salzberg SL: **A whole-genome assembly of the domestic cow, Bos taurus**. *Genome Biol* 2009, **10**:R42.

2. Quackenbush J, Cho J, Lee D, Liang F, Holt I, Karamycheva S, Parvizi B, Pertea G, Sultana R, White J: **The TIGR Gene Indices: analysis of gene transcript sequences in highly sampled eukaryotic species**. *Nucl. Acids Res.* 2001, **29**:159–164.

3. Gremme G, Brendel V, Sparks ME, Kurtz S: **Engineering a software tool for gene structure prediction in higher organisms**. *Information and Software Technology* 2005, **47**:965–978.

4. Lewis SE, Searle SMJ, Harris N, Gibson M, Lyer V, Richter J, Wiel C, Bayraktaroglir L, Birney E, Crosby MA, Kaminker JS, Matthews BB, Prochnik SE, Smithy CD, Tupy JL, Rubin GM, Misra S, Mungall CJ, Clamp ME: **Apollo: a sequence annotation editor**. *Genome Biol.* 2002, **3**:RESEARCH0082.

Gene: ACAN
Chr (strand): 21 (+)
txStart: 20800157
txEnd: 20868837
cdsStart: 20829111
cdsEnd: 20868734
exons: 17
exonStarts: 20800157, 20829104, 20831451, 20833151, 20834629, 20835037, 20836594, 20839216, 20840178, 20841381, 20844357, 20847583, 20865291, 20865854, 20867022, 20867969, 20868658
exonEnds: 20800509, 20829180, 20831834, 20833325, 20834756, 20835330, 20836998, 20839390, 20840305, 20841674, 20844572, 20851653, 20865449, 20865936, 20867166, 20868151, 20868837

Gene: APAF1
Chr (strand): 5 (-)
txStart: 63125314
txEnd: 63206764
cdsStart: 63125314
cdsEnd: 63206764
exons: 26

exonStarts: 63125177, 63125482, 63126311, 63137636, 63139812, 63140013, 63144867, 63145509, 63146886, 63149720, 63150274, 63154793, 63156193, 63159487, 63161933, 63170426, 63175619, 63178936, 63181417, 63183565, 63185578, 63192120, 63192542, 63198557, 63200442, 63206764
exonEnds: 63125314, 63125638, 63126508, 63137819, 63139924, 63140144, 63145105, 63145676, 63147017, 63149833, 63150458, 63154919, 63156318, 63159618, 63162058, 63170587, 63175747, 63179061, 63181536, 63183681, 63185703, 63192239, 63192667, 63198682, 63200588, 63207077

Gene: ATP2A1
Chr (strand): 25 (-)
txStart: 26204671
txEnd: 26187372
cdsStart: 26204482
cdsEnd: 26188119
exons: 22
exonStarts: 26204365, 26203498, 26201977, 26200943, 26198520, 26197566, 26197133, 26196535, 26193975, 26193588, 26193224, 26191637, 26191294, 26190149, 26189730, 26189419, 26189160, 26188936, 26188694, 26188436, 26188079, 26187372
exonEnds: 26204671, 26203580, 26202081, 26201081, 26198600, 26197651, 26197430, 26196701, 26194063, 26193690, 26193355, 26191759, 26191512, 26190484, 26189950, 26189621, 26189245, 26189069, 26188811, 26188553, 26188123, 26187685

Gene: B2M
Chr (strand): 10 (-)
txStart: 104139184
txEnd: 104143771
cdsStart: 104139184
cdsEnd: 104143771
exons: 3
exonStarts: 104139093, 104142970, 104143771
exonEnds: 104139184, 104143245, 104145314

Gene: BCKDHA
Chr (strand): 18 (+)
txStart: 50819286
txEnd: 50838369
cdsStart: 50819376
cdsEnd: 50838020
exons: 10
exonStarts: 50819286, 50819371, 50828838, 50829130, 50832646, 50834705, 50836114, 50836418, 50836772, 50837850
exonEnds: 50819308, 50819489, 50829023, 50829216, 50832754, 50834866, 50836320, 50836559, 50836943, 50838369

Gene: CRIM1
Chr (strand): 11 (+)
txStart: 18834698
txEnd: 19043656

cdsStart: 18835047
cdsEnd: 19041796
exons: 17
exonStarts: 18834698, 18876350, 18928344, 18929665, 18952089, 18961646, 18967407, 18987946, 18996344, 18999210, 18999839, 19003610, 19009194, 19029657, 19037537, 19040118, 19041620
exonEnds: 18835377, 18876523, 18928586, 18929785, 18952210, 18961828, 18967604, 18988074, 18996500, 18999331, 19000048, 19003825, 19009415, 19029851, 19037656, 19040305, 19043656

Gene: CSN2
Chr (strand): 6 (-)
txStart: 87188004
txEnd: 87179499
cdsStart: 87186007
cdsEnd: 87180588
exons: 9
exonStarts: 87187976, 87185957, 87185203, 87185064, 87183147, 87183014, 87181195, 87180552, 87179499
exonEnds: 87188004, 87186019, 87185229, 87185090, 87183170, 87183055, 87181692, 87180593, 87179821

Gene: DCX
Chr (strand): X (-)
txStart: 64861454
txEnd: 64741273
cdsStart: 64860705
cdsEnd: 64742327
exons: 7
exonStarts: 64861065, 64860342, 64850230, 64772852, 64770736, 64755934, 64741273
exonEnds: 64861454, 64860727, 64850570, 64772954, 64770873, 64756028, 64742383

Gene: DGAT1
Chr (strand): 14 (+)
txStart: 1795425
txEnd: 1804838
cdsStart: 1795438
cdsEnd: 1804540
exons: 14
exonStarts: 1795425, 1799245, 1801276, 1801396, 1801574, 1801842, 1802037, 1802251, 1802396, 1802590, 1803951, 1804103, 1804272, 1804407
exonEnds: 1795628, 1799332, 1801316, 1801481, 1801626, 1801947, 1802150, 1802325, 1802499, 1802628, 1804016, 1804190, 1804334, 1804838

Gene: EPS8
Chr (strand): 5 (-)
txStart: 94467480
txEnd: 94447013
cdsStart: 94466303

cdsEnd: 94448652
exons: 12
exonStarts: 94466985, 94466179, 94458419, 94457535, 94455264, 94454563, 94450854, 94448728, 94448651, 94448420, 94448276, 94447013
exonEnds: 94467480, 94466314, 94458500, 94457607, 94455360, 94454700, 94450990, 94448877, 94448656, 94448430, 94448341, 94447573

Gene: EVC2
Chr (strand): 6 (+)
txStart: 105330747
txEnd: 105451901
cdsStart: 105330834
cdsEnd: 105451674
exons: 15
exonStarts: 105330747, 105336587, 105366953, 105373754, 105377769, 105380854, 105389397, 105394391, 105401116, 105420716, 105429588, 105431683, 105437050, 105448810, 105451404
exonEnds: 105330881, 105336726, 105367277, 105373993, 105377944, 105381013, 105389851, 105394595, 105401238, 105420943, 105429802, 105431770, 105437246, 105448911, 105451901

Gene: F11
Chr (strand): 27 (+)
txStart: 15350937
txEnd: 15370082
cdsStart: 15352136
cdsEnd: 15369432
exons: 15
exonStarts: 15350937, 15352135, 15354679, 15356121, 15357119, 15358901, 15359719, 15362156, 15362359, 15362611, 15365705, 15366947, 15367918, 15368566, 15369271
exonEnds: 15351277, 15352190, 15354841, 15356227, 15357278, 15359010, 15359878, 15362265, 15362521, 15362717, 15365873, 15367122, 15368013, 15368705, 15370082

Gene: FANCI
Chr (strand): 21 (+)
txStart: 21137918
txEnd: 21198618
cdsStart: 21137918
cdsEnd: 21198015
exons: 37
exonStarts: 21137918, 21143792, 21148879, 21149435, 21149672, 21151169, 21151533, 21152065, 21153680, 21157108, 21157868, 21160931, 21162477, 21168317, 21168907, 21169769, 21171485, 21175328, 21178994, 21180222, 21180469, 21181103, 21181843, 21185396, 21185897, 21186937, 21189160, 21190043, 21190270, 21190524, 21190896, 21192492, 21192626, 21194926, 21196529, 21196916, 21197935
exonEnds: 21138001, 21143864, 21149009, 21149591, 21149729, 21151210, 21151656, 21152150, 21153806, 21157197, 21158004, 21161111, 21162564, 21168447, 21168977, 21169883, 21171607, 21175396, 21179095, 21180392, 21180590, 21181267, 21182022, 21185562, 21185973, 21187053, 21189211, 21190170, 21190338, 21190617, 21191083, 21192545, 21192685, 21194994, 21196624,

21197020, 21198618

Gene: FASN
Chr (strand): 19 (+)
txStart: 51384922
txEnd: 51403614
cdsStart: 51386877
cdsEnd: 51403038
exons: 42
exonStarts: 51384922, 51386866, 51387774, 51389276, 51389689, 51390003, 51390211, 51390417, 51391235, 51391796, 51392081, 51392874, 51393108, 51393348, 51393659, 51394011, 51394264, 51394537, 51394733, 51395082, 51395339, 51396129, 51396738, 51397214, 51397497, 51397696, 51398001, 51398362, 51398598, 51398867, 51399182, 51399430, 51399738, 51400065, 51400327, 51400618, 51400855, 51401430, 51401844, 51402166, 51402466, 51402649
exonEnds: 51385000, 51387003, 51387926, 51389449, 51389889, 51390125, 51390326, 51390551, 51391697, 51391983, 51392270, 51392968, 51393242, 51393551, 51393774, 51394183, 51394455, 51394617, 51394921, 51395261, 51395542, 51396427, 51397130, 51397378, 51397615, 51397850, 51398204, 51398512, 51398776, 51398986, 51399304, 51399653, 51399939, 51400216, 51400418, 51400769, 51401097, 51401618, 51402074, 51402386, 51402564, 51403614

Gene: FUT4
Chr (strand): 15(-)
txStart: 16243413
txEnd: 16241316
cdsStart: 16243368
cdsEnd: 16242172
exons: 1
exonStarts: 16241316
exonEnds: 16243413

Gene: KDSR
Chr (strand): 24 (-)
txStart: 62144321
txEnd: 62118140
cdsStart: 62144294
cdsEnd: 62118580
exons: 6
exonStarts: 62144226, 62138749, 62129306, 62126321, 62122532, 62118140
exonEnds: 62144321, 62138940, 62129389, 62126404, 62122633, 62118699

Gene: KITLG
Chr (strand): 5 (-)
txStart: 18420108
txEnd: 18317428
cdsStart: 18420024
cdsEnd: 18326596
exons: 9

exonStarts: 18420010, 18377331, 18363202, 18342815, 18339747, 18328638, 18327626, 18326559, 18317428
exonEnds: 18420108, 18377444, 18363264, 18342985, 18339906, 18328747, 18327693, 18326635, 18317795

Gene: KRT5
Chr (strand): 5 (+)
txStart: 27541284
txEnd: 27547603
cdsStart: 27541428
cdsEnd: 27547238
exons: 9
exonStarts: 27541284, 27542548, 27543524, 27543695, 27544400, 27544913, 27545268, 27546415, 27546922
exonEnds: 27541985, 27542762, 27543584, 27543790, 27544564, 27545038, 27545488, 27546449, 27547603

Gene: LRP4
Chr (strand): 15 (-)
txStart: 77716107
txEnd: 77663790
cdsStart: 77715842
cdsEnd: 77664021
exons: 38
exonStarts: 77715833, 77701062, 77699548, 77698874, 77698375, 77697909, 77697547, 77696795, 77696071, 77695737, 77695242, 77693965, 77692965, 77691640, 77691303, 77690798, 77688717, 77688461, 77687581, 77686636, 77684984, 77684768, 77683717, 77683286, 77683008, 77681948, 77681629, 77680948, 77679055, 77678776, 77677645, 77675816, 77675429, 77674555, 77669812, 77668895, 77667136, 77663790
exonEnds: 77716107, 77701220, 77699664, 77698987, 77698491, 77698037, 77697666, 77696920, 77696196, 77695871, 77695367, 77694195, 77693121, 77691857, 77691479, 77690920, 77688925, 77688542, 77687686, 77686837, 77685173, 77684899, 77683857, 77683372, 77683179, 77682110, 77681854, 77681251, 77679273, 77678910, 77677753, 77675960, 77675542, 77674690, 77669879, 77668982, 77667277, 77664353

Gene: LTF
Chr (strand): 22 (+)
txStart: 53522006
txEnd: 53557385
cdsStart: 53522045
cdsEnd: 53555898
exons: 18
exonStarts: 53522006, 53526609, 53529749, 53530153, 53530952, 53532043, 53537446, 53538052, 53538752, 53540863, 53543180, 53543850, 53545916, 53550815, 53552803, 53554118, 53555864, 53557284
exonEnds: 53522087, 53526772, 53529857, 53530335, 53531099, 53532098, 53537624, 53538226, 53538906, 53540953, 53543227, 53544005, 53546057, 53550882, 53552987, 53554307, 53556096,

53557385

Gene: LYST
Chr (strand): 28 (-)
txStart: 8567662
txEnd: 8422583
cdsStart: 8567655
cdsEnd: 8424445
exons: 51
exonStarts: 8567464, 8552189, 8548496, 8546042, 8544347, 8542409, 8539938, 8539101, 8533708, 8531555, 8530274, 8528700, 8523686, 8522656, 8520288, 8515323, 8514262, 8510218, 8508557, 8502596, 8499833, 8498960, 8497088, 8495498, 8493164, 8492348, 8488477, 8486629, 8484258, 8479843, 8479194, 8478860, 8476679, 8476496, 8475671, 8473786, 8471590, 8465053, 8458780, 8457280, 8454046, 8450731, 8444293, 8442601, 8439014, 8436531, 8430885, 8430510, 8426664, 8425508, 8422583
exonEnds: 8567662, 8552279, 8550575, 8547065, 8544508, 8542565, 8540164, 8539167, 8533817, 8531972, 8530418, 8528873, 8523846, 8522846, 8520533, 8515496, 8514411, 8510355, 8508755, 8502727, 8500460, 8499137, 8497257, 8495728, 8493330, 8492500, 8488668, 8486807, 8484464, 8480019, 8479270, 8479048, 8476824, 8476592, 8475732, 8473841, 8471742, 8465297, 8458846, 8457436, 8454186, 8450948, 8444523, 8442790, 8439150, 8436629, 8431024, 8430607, 8426820, 8425579, 8424583

Gene: MGST1
Chr (strand): 5 (-)
txStart: 93946277
txEnd: 93926635
cdsStart: 93942180
cdsEnd: 93927151
exons: 4
exonStarts: 93946180, 93942055, 93939150, 93926635
exonEnds: 93946277, 93942195, 93939244, 93927397

Gene: MITF
Chr (strand): 22 (-)
txStart: 31839314
txEnd: 31735782
cdsStart: 31839314
cdsEnd: 31736217
exons: 10
exonStarts: 31839259, 31826415, 31768019, 31766884, 31764658, 31754241, 31752215, 31746439, 31742357, 31735782
exonEnds: 31839314, 31826664, 31768246, 31766967, 31764753, 31754358, 31752289, 31746514, 31742504, 31736618

Gene: MOCS1
Chr (strand): 23 (-)
txStart: 13866995

txEnd: 13832469
cdsStart: 13866914
cdsEnd: 13833191
exons: 11
exonStarts: 13866801, 13858301, 13856667, 13843066, 13840653, 13840252, 13839729, 13839139, 13836649, 13836096, 13832469
exonEnds: 13866995, 13858427, 13856834, 13843230, 13840714, 13840363, 13839841, 13839249, 13836769, 13836143, 13833951

Gene: PITPNM3
Chr (strand): 19 (+)
txStart: 25933697
txEnd: 26018336
cdsStart: 25933697
cdsEnd: 26017902
exons: 20
exonStarts: 25933697, 25940042, 25942789, 25977407, 25994161, 25994791, 25998211, 25998802, 25999550, 26003160, 26004574, 26006535, 26007265, 26009297, 26012295, 26012708, 26013139, 26015180, 26015776, 26017600
exonEnds: 25933795, 25940084, 25942884, 25977454, 25994237, 25995026, 25998400, 25998924, 25999734, 26003332, 26004744, 26006726, 26007413, 26009413, 26012411, 26012856, 26013288, 26015363, 26015904, 26018336

Gene: PLAG1
Chr (strand): 14 (-)
txStart: 25052394
txEnd: 25007082
cdsStart: 25008544
cdsEnd: 25007291
exons: 3
exonStarts: 25052215, 25012350, 25007082
exonEnds: 25052394, 25012448, 25008548

Gene: PLCB3
Chr (strand): 29 (+)
txStart: 43170291
txEnd: 43188249
cdsStart: 43170432
cdsEnd: 43187727
exons: 31
exonStarts: 43170291, 43173826, 43174172, 43174329, 43174691, 43174875, 43175021, 43175199, 43175830, 43176071, 43177123, 43177564, 43177724, 43178637, 43179658, 43180668, 43181035, 43181502, 43181737, 43182569, 43182755, 43183730, 43185088, 43185343, 43185490, 43186237, 43186493, 43186808, 43187013, 43187357, 43187524
exonEnds: 43170530, 43173903, 43174240, 43174469, 43174770, 43174928, 43175096, 43175299, 43175996, 43176217, 43177363, 43177648, 43177910, 43178842, 43179754, 43180752, 43181159, 43181656, 43181898, 43182668, 43182859, 43183821, 43185253, 43185378, 43185682, 43186393,

43186569, 43186921, 43187070, 43187443, 43188249

Gene: PLD4
Chr (strand): 21 (+)
txStart: 70995733
txEnd: 71004743
cdsStart: 70997394
cdsEnd: 71004475
exons: 11
exonStarts: 70995733, 70997228, 70999611, 71000430, 71000797, 71001120, 71002072, 71002912, 71003759, 71003864, 71004273
exonEnds: 70995782, 70997420, 70999810, 71000613, 71000917, 71001247, 71002272, 71003051, 71003789, 71003960, 71004743

Gene: PRKG2
Chr (strand): 6 (-)
txStart: 97735624
txEnd: 97651497
cdsStart: 97735536
cdsEnd: 97652569
exons: 17
exonStarts: 97735458, 97729722, 97728193, 97724550, 97716146, 97713677, 97710654, 97706403, 97704768, 97702742, 97699715, 97697077, 97672666, 97667320, 97665516, 97654843, 97651497
exonEnds: 97735624, 97729835, 97728298, 97724613, 97716223, 97713771, 97710722, 97706501, 97704921, 97702878, 97699804, 97697218, 97672829, 97667442, 97665578, 97654909, 97652664

Gene: SPEM1
Chr (strand): 19 (+)
txStart: 27733908
txEnd: 27735192
cdsStart: 27733917
cdsEnd: 27735158
exons: 3
exonStarts: 27733908, 27734160, 27734419
exonEnds: 27734060, 27734217, 27735192

Gene: TG
Chr (strand): 14 (-)
txStart: 9508938
txEnd: 9262209
cdsStart: 9508896
cdsEnd: 9262209
exons: 47
exonStarts: 9508830, 9507787, 9507259, 9505688, 9504888, 9495978, 9495377, 9495008, 9487632, 9486462, 9485978, 9483686, 9483000, 9482340, 9480029, 9478786, 9474909, 9473480, 9472031, 9469709, 9461819, 9459539, 9456049, 9455409, 9453584, 9448252, 9443775, 9434313, 9432971, 9432149, 9430852, 9428788, 9427410, 9426338, 9418155, 9396262, 9393926, 9388175, 9386505,

9384346, 9376981, 9314427, 9312812, 9298761, 9296197, 9263796, 9262209
exonEnds: 9508938, 9507895, 9507356, 9505891, 9505047, 9496084, 9495520, 9495193, 9488732, 9487049, 9486217, 9483823, 9483077, 9482452, 9480131, 9478986, 9475121, 9473634, 9472187, 9469933, 9461968, 9459709, 9456165, 9455524, 9453692, 9448443, 9443939, 9434378, 9433051, 9432286, 9431028, 9428899, 9427489, 9426481, 9418217, 9396396, 9394090, 9388394, 9386598, 9384505, 9377183, 9314591, 9312979, 9298942, 9296304, 9263930, 9262440

Gene: TMEM95
Chr (strand): 19 (+)
txStart: 27688383
txEnd: 27689818
cdsStart: 27688399
cdsEnd: 27689818
exons: 7
exonStarts: 27688383, 27688794, 27689045, 27689227, 27689380, 27689576, 27689791
exonEnds: 27688567, 27688850, 27689125, 27689247, 27689460, 27689663, 27689818
